# Perceived Versus Performance Fatigability in Patients With Rheumatoid Arthritis

**DOI:** 10.3389/fphys.2018.01395

**Published:** 2018-10-10

**Authors:** Kristina Marrelli, Arthur J. Cheng, Julie D. Brophy, Geoffrey A. Power

**Affiliations:** ^1^Department of Human Health and Nutritional Sciences, College of Biological Sciences, University of Guelph, Guelph, ON, Canada; ^2^Department of Physiology and Pharmacology, Karolinska Institutet, Stockholm, Sweden; ^3^Community Rheumatologist, Wellington Ortho and Rehab, Guelph, ON, Canada

**Keywords:** inflammation, rheumatoid arthritis, performance fatigability, perceived fatigability, fatigue, fatigability, cytokines

## Abstract

Rheumatoid arthritis (RA) is a chronic, inflammatory disease that affects 1% of the general population. Fatigue is a common complaint of patients with RA, however their perceived fatigue may be more exacerbated than objective measures of fatigue may indicate. The assessment of fatigue is made complex due to inconsistent and vague terms used to define fatigue, and the task dependence of fatigability. Fatigue is defined as a state of exhaustion and decreased strength, while fatigability indicates an individual’s susceptibility to fatigue. In order to offer some clarity to the manifestation of fatigue in clinical populations, in this review we outline that fatigue should be described with subsections that are related to the symptom, such as: perceived fatigability and performance fatigability. Where perceived fatigability indicates the subjective state of the individual and thus involves the individual’s subjective measure of fatigue, performance fatigability would be measured through clinical and laboratory-based assessments that quantify the functional decline in performance. This review describes RA and the various neuromuscular changes associated with the disease that can lead to alterations in both perceived and performance fatigue. From there, we discuss fatigue and RA, how fatigue can be assessed, effects of exercise interventions on RA symptoms and fatigue, and recommendations for future studies investigating subjective and objective measures of fatigability.

Rheumatoid arthritis (RA) is a chronic, inflammatory disease that affects many individuals regardless of their demographic (Veldhuijzen Van Zanten et al., 2015). Fatigue, or the state of exhaustion and increased acute weakness, is a common symptom reported by 40–80% of individuals with RA and its co-morbidities: rheumatoid cachexia and sarcopenia (Cooney et al., 2011; Nikolaus et al., 2013; Louati and Berenbaum, 2015). Attempts have been made to study fatigue within the rheumatoid population, yet there are still considerable knowledge gaps regarding the underlying mechanisms of fatigue. Furthermore, in RA populations, a commonly accepted definition of fatigue does not exist (Nikolaus et al., 2013). Amongst primary care providers, there has been a great deal of confusion regarding the topic of fatigue and the various types of fatigue that individuals experience possibly because terminology surrounding ‘fatigue’ is fairly ambiguous – ranging from general malaise and tiredness to transient impairments in neuromuscular function and muscle force production. The multifactorial etiology of fatigue has driven researchers to describe fatigue based on its likely origin; however, the descriptions have been depicted as too unclear and have only led to confusion regarding clinical populations (Enoka and Duchateau, 2016).

Owing to the multiple origins of fatigue, it has been difficult to determine the best ways to measure fatigue in clinical populations. Moreover, there has been confusion regarding whether or not self-reported measures of fatigue reflect laboratory-based objective measures (do Espírito Santo et al., 2017). Some studies assess only the psychological factors that contribute to feelings of fatigue and tiredness (Hewlett et al., 2011) while others assess the specific muscular, peripheral, and central nervous system impairments reducing neuromuscular performance (Enoka and Duchateau, 2016). In order to grasp a true understanding of fatigue in the rheumatoid population, it is critical for researchers and clinicians to assess both perceived and performance fatigability and draw parallels between these findings. The purpose of this review is to explore the relationship between RA and fatigue, the different types of fatigue that have been explored in literature, the measures of fatigue that are used to assess both performance and perceived fatigue, what clinicians and researchers are recommending to manage the symptoms of RA, and lastly, future directions of evaluating fatigue in RA populations.

## Rheumatoid Arthritis

Rheumatoid arthritis (RA) is a chronic, multi-system, autoimmune disease that affects approximately 1% of the general population (Veldhuijzen Van Zanten et al., 2015). Women are more commonly targeted with a peak onset between the ages of 40 and 60 years of age; however, RA can affect individuals of all demographics (Londhe and Guttridge, 2015). It is a debilitating disease that is often characterized by joint pain, swelling, and stiffness due to persistent inflammation (Londhe and Guttridge, 2015; Veldhuijzen Van Zanten et al., 2015). Sustained synovitis can lead to permanent structural damage, which in turn reduces the everyday functionality of patients with RA (Cooney et al., 2013; Londhe and Guttridge, 2015).

The synovial membranes of individuals with RA are often characterized by increased vascularity and intrusion of inflammatory cells, which are suspected to be the key mediators of the disease (Londhe and Guttridge, 2015). There is an approximate 3–100 fold increase in the levels of pro-inflammatory cytokines, specifically interleukin-6 (IL-6), tumor necrosis factor alpha (TNF-a), and interleukin 1-B (IL-1B) (Cooney et al., 2011). IL-6 perpetuates RA by stimulating the secretion of vascular endothelial growth factor (VEGF), enhancing angiogenesis in the synovium. IL-6 also works synergistically with IL-1B to increase the production of matrix metalloproteinases (MMPs), leading to joint and cartilage destruction (Londhe and Guttridge, 2015). TNF-a and IL-1 are potent stimulators of mesenchymal cells that release matrix MMPs, which are involved in degradation of tissue and prevent production of tissue inhibitors of metalloproteinases (TIMPs) (Londhe and Guttridge, 2015). Increased catabolism of muscle tissue coinciding with the downregulation of anabolic factors that accompany RA, such as insulin-like growth factor (IGF-1), drastically alters muscle-protein turnover to a net catabolic state (Cooney et al., 2011; Dogan et al., 2015; Londhe and Guttridge, 2015). Increased net catabolism causes an increase in resting energy expenditure (REE), efflux of amino acids from the muscles to the liver, and an increase in acute phase reactants, fibrinogen, and C-reactive protein (CRP) (Londhe and Guttridge, 2015).

A hypothetical model of the disablement process in patients with RA was proposed (Balsamo et al., 2014), suggesting there are many relationships between multiple variables that will impact the disability levels in RA. The main disease-disability pathway follows a series of stages including pathology, impairment, functional limitation, and lastly, disability (Balsamo et al., 2014). Inflammation and long-term joint damage lead to symptoms of impairment, including muscle pain and weakness as well as self-reported fatigue, resulting in functional limitations in mobility, strength and dexterity (Escalante and Del Rincón, 2002; Alomari et al., 2012). These limitations lead to physical disability and, ultimately, cause patients to suffer a reduced quality of life (Alomari et al., 2012).

## Sarcopenia and Rheumatoid Cachexia: Co-Morbidities of RA

Chronic inflammatory diseases such as RA have been linked with various co-morbidities such as rheumatoid cachexia and sarcopenia Matschke et al. (2010a). Both conditions lead to muscle wasting and may influence fatigability in RA patients (Cooney et al., 2011). Rheumatoid cachexia is reported in approximately two-thirds of the RA population (Cooney et al., 2011). This condition presents as an accelerated loss of lean muscle mass while maintaining a normal or increased fat mass, contributing to disability and a poorer quality of life (Cooney et al., 2011). The total mass associated with muscle and bone within the body, known as body cell mass (BCM), was decreased by 13% in adults with RA compared to controls (Roubenoff et al., 1994). The loss of BCM was primarily caused by cytokine-mediated catabolism, a decrease in physical activity, inadequate nutrition and effects of medication (Roubenoff et al., 1994). However, the fundamental feature of rheumatoid cachexia appears to be an excess of the pro-inflammatory cytokines that are involved in RA (TNF-a, IL-1B, IL-6, and IFN-y) which leads to increased muscle wasting and protein degradation (Cooney et al., 2011; Masuko, 2014; Londhe and Guttridge, 2015). In older patients, in addition to RA cachexia, muscle loss can also occur through the process known as sarcopenia. Sarcopenia is a condition currently defined as “an age-related loss of functional quality (of muscle) in addition to muscle weakness and muscle protein mass loss” (Power et al., 2013; Dogan et al., 2015) While sarcopenia is classically associated with aging, it is believed that accelerated muscle loss in RA patients occurs due to chronic inflammation and the effects that are associated with sarcopenia (Masuko, 2014). The primary driver of age-related muscle wasting appears to be a loss of functional motor units (α motor neuron and the muscle fibers it innervates). Following the death of a parent motor neuron, the muscle fibers previously innervated are orphaned, and although some are recaptured via collateral reinnervation, whole muscle atrophy is inevitable. (Power et al., 2014; Power et al., 2016). Additionally, increased inflammatory mediators, particularly cytokines, and other immunological changes due to aging may contribute to the loss of lean muscle mass (Dogan et al., 2015). Therefore, it has been proposed that changes to the inflammatory environment and lifestyle modifications accompanying RA, such as increased cytokine levels and reduced physical activity due to immobilization from pain, stiffness, and joint damage, accelerate the progression and the occurrence of sarcopenia in the RA population (Dogan et al., 2015; Krajewska-Wlodarczyk, 2016). Both rheumatoid cachexia and sarcopenia are involved in a vicious cycle of disease progression in RA patients, causing further deleterious effects. It has been demonstrated that exercise as a form of therapy is very beneficial (discussed below) in reducing the progression and risk of sarcopenia and rheumatoid cachexia, as well as alleviating some of the physical limitations commonly experienced by patients with RA (Cooney et al., 2011). These co-morbidities can be reduced and safely managed through the completion of regular physical activity (Cooney et al., 2011).

## Neuromuscular Changes in RA Patients

Histopathological analysis of muscle from patients with stable RA [stable disease activity due to no flare or change in medication for the previous 3 months (Matschke et al., 2010b)] tend to report characteristics of ‘normal’ muscle (Lundberg and Nader, 2008). However, even with similar whole muscle properties, in two-thirds of patients with RA there is often a 25–50% reduction in muscle strength (Stenstrom and Minor, 2003). Some human trials reveal muscle weakness is often a consequence of skeletal muscle mass loss as opposed to changes in muscle quality (i.e., force/contractile properties) or voluntary activation (Matschke et al., 2010b). In contrast, other studies suggest that muscle mass loss cannot account for the decrease in muscle strength alone (Helliwell and Jackson, 1994), proposing that the contractile function of muscles affected by RA are impaired, thus causing muscle weakness (de Palma et al., 2000; Yamada et al., 2009; Yamada et al., 2015). Therefore, muscle weakness in patients with RA may be driven by two major contributors: the loss of lean muscle mass (Matschke et al., 2010b) and a reduction in intrinsic muscle quality due to impaired muscle contractility (de Palma et al., 2000; Yamada et al., 2009).

Excitation-contraction (EC) coupling is the physiological process of converting a neuronal stimulus from the central nervous system to a mechanical response that leads to muscle contraction and consequently force production (Allen et al., 2008). Specifically, EC coupling occurs when depolarization of the sarcolemma results in increased sarcoplasmic reticulum (SR) Ca^2+^ release into the myoplasm (Allen et al., 2008), causing force generation when the myofibrillar proteins actin and myosin interact to form crossbridges. Impaired EC coupling may be the critical contributor to intrinsic muscle weakness in RA (Yamada et al., 2015). For instance, structural damage in the myofibrillar proteins and sarcotubular systems (de Palma et al., 2000) are commonly observed in muscle biopsies of RA patients with symptoms of muscle weakness (Russell and Hanna, 1988). Chronically elevated redox stress may underlie the pathology as nitrosative modifications of the myofibrillar protein observed in the muscle biopsies of RA patients as demonstrated within an animal model of RA (Yamada et al., 2015). Using the collagen-induced arthritis (CIA) mouse model that demonstrates similar pathological characteristics of human RA, Yamada et al. (2015) presented an apparent decrease in muscle force per cross-sectional area of the soleus muscle. Reduced force production was complemented with slower force development and slowed relaxation, a decreased maximal shortening velocity, and modifications in myofibrillar proteins induced by the highly reactive nitrogen species, peroxynitrite. The authors hypothesized that an increase in peroxynitrite-derived radical generation in rheumatoid muscle impaired cross-bridge cycling (Yamada et al., 2009). High levels of radicals, such as peroxynitrate and other reactive oxygen and nitrogen species (ROS/RNS), are associated with damage to muscle cellular components and contractile dysfunction in chronic diseases (Yamada et al., 2015). Despite an increased Ca^2+^ level during tetanic contractions, there was a decreased force per cross-sectional area in individual CIA muscle fibers, indicating weakness compared to control mice (Yamada et al., 2015). Overall, these studies demonstrated that contractile dysfunction is associated with decreased myofibrillar Ca^2+^ sensitivity, rather than an impaired excitability, SR Ca^2+^ uptake, or lower concentrations of cytosolic Ca^2+^ within animal models of RA (Yamada et al., 2009, 2015).

Decreased myofibrillar Ca^2+^ sensitivity indicates that muscles need a much higher concentration of Ca^2+^ released in order to function (i.e., produce the same level of force) at the same level as healthy muscle (Yamada et al., 2015). A potential consequence of the higher Ca^2+^ concentration for force generation is an increased energy cost of contraction due to elevated ATP consumption by the sarcoplasmic reticulum Ca^2+^-ATPase to pump Ca^2+^ back into the sarcoplasmic reticulum; potentially exacerbating muscle fatigue. At the whole human level, the required release of additional Ca^2+^ to achieve the same force level as healthy individuals requires increased motor unit activation. The additional descending cortical drive to the motor unit pool potentially increases an RA patients’ perception of effort required to achieve a given force level (Pageaux, 2016). Increased self-reported perception of effort can therefore have a significant effect on an individual’s fatigue status, exercise intolerance, and lead to further complications in RA (Matschke et al., 2010b; Pageaux, 2016).

In summary, while animal data demonstrates that the major peripheral mediators of exercise-induced weakness are decreased myofibrillar Ca^2+^ sensitivity and increased intracellular Ca^2+^ concentration during muscle contraction, data from human muscle biopsies also indicate intrinsic contractile dysfunction and thus, decreased muscle strength in patients with RA (de Palma et al., 2000). Therefore, neuromuscular changes associated with RA are speculated to be involved with, and lead to, one of RA’s most prominent symptoms: fatigue.

## Fatigue and RA

**FIGURE 1:**
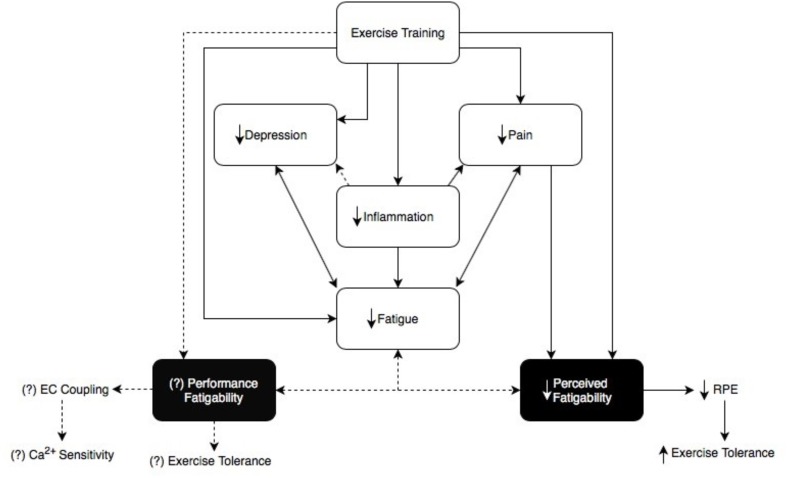
This figure demonstrates the link between fatigue, depression, pain, and inflammation (Nikolaus et al., 2013; Louati and Berenbaum, 2015). Fatigue in RA can be subdivided into ‘Perceived fatigability’ and ‘Performance fatigability’, each with different underlying mechanisms (Enoka and Duchateau, 2016). Exercise training shows a speculative relationship between perceived and performance fatigability (Cooney et al., 2011; Veldhuijzen Van Zanten et al., 2015). This diagram explains that not only does exercise training reduce inflammation and alleviate symptoms in RA patients, it may also help to decrease performance and perceived fatigability which may ultimately act upon and reduce one another as well (Cooney et al., 2011; Veldhuijzen Van Zanten et al., 2015). We propose there is a distinct relationship between exercise training and its effects on performance and perceived fatigability relating back to the overall symptom of fatigue in this clinical population. Dotted lines represent the speculative relationship between exercise training and performance fatigability, as well as performance fatigability and perceived fatigability.

Fatigue is a common symptom reported by patients with RA (Louati and Berenbaum, 2015). Approximately 40–80% of patients with RA experience fatigue, with 50% of individuals reporting severe fatigue (Nikolaus et al., 2013; Louati and Berenbaum, 2015). Medications used as treatment for RA, such as methotrexate, can induce or amplify perceived fatigue in patients with RA (Kluger et al., 2013), further complicating assessments of fatigue in RA patients. Clinically, a generally accepted definition of fatigue has yet to be defined, but some individuals have described it as “a state of exhaustion and decreased strength accompanied by a feeling of weariness, sleepiness and irritability, with a cognitive component” (Louati and Berenbaum, 2015). Fatigue is a valid outcome measure of RA, as well as a reliable differentiator of quality of life between individuals with RA who are doing poorly and those who are doing well (Balsamo et al., 2014). There is clear evidence demonstrating a common link between inflammation and fatigue, depression, and pain - the three most expressed symptoms associated with RA (**Figure 1**) (Louati and Berenbaum, 2015). Although each possesses complex mechanisms of action, it has been documented that cytokines are associated with all three domains (Louati and Berenbaum, 2015). Thus, the link between the three may potentially be inflammation associated with RA. Some studies have even demonstrated higher levels of IL-1 in the cerebrospinal fluid of individuals with RA compared to controls which correlated with their intensity of fatigue (Louati and Berenbaum, 2015). Furthermore, depressive symptoms and severity are exacerbated by increased levels of pro-inflammatory cytokines IL-1B, IL6, IL-18, TNF-α, and CRP (Slowik et al., 2017). Fibromyalgia, a condition characterized by chronic widespread pain is also common in RA and can lead to similar symptoms of RA such as depression and fatigue (Dhir et al., 2009), yet this specific condition is beyond the scope of this literature review. Because fatigue is often associated with psychological factors such as depression (Hewlett et al., 2010; Enoka and Duchateau, 2016), it can be important to separate whether fatigue is directly or indirectly related to disease progression in RA. Nonetheless, it cannot be ruled out that fatigue has a central origin as it has been shown that rest does not improve perceived symptoms of fatigue in chronic inflammatory diseases (Staud, 2012).

## Fatigue and Fatigability

Fatigue is distinguishable from other symptoms established in various inflammatory or neurological diseases, such as general malaise, depression, and apathy (Kluger et al., 2013). There have been multiple adjectives attached to the term “fatigue” including central, peripheral, supraspinal, muscle as defined in **Table 1**.

**Table 1:** Definitions of the multiple subsets of fatigue.

Fatigue	“A state of exhaustion and decreased strength accompanied by a feeling of weariness, sleepiness and irritability, with a cognitive component” (Enoka and Duchateau, 2016)
Central fatigue	“Difficulty initiating or sustaining voluntary activities, leading to a progressive reduction in the activation of the muscle” (Gandevia, 2001; Staud, 2012)
Peripheral fatigue	“Fatigue produced by changes at or distal to the neuromuscular junction” (Gandevia, 2001)
Supraspinal fatigue	“A subset of central fatigue produced by failure to generate output from the motor cortex” (Gandevia, 2001)
Muscle fatigue	“A motor deficit, perception or a decline in […] function, leading to a gradual decrease in the force capacity of muscle or the endpoint of a sustained activity” (Enoka and Duchateau, 2008)
Pathological fatigue	“Fatigue experienced as a symptom/outcome in acute or chronic diseases, such as RA, that often does not improve with rest” (Enoka and Duchateau, 2016)

The terms “central” and “peripheral” indicate the most probable origin in the body that contributes to fatigue (Enoka and Duchateau, 2016). In some literature, central fatigue is described as “progressive reduction in voluntary activation of muscle during exercise” (Gandevia, 2001) where peripheral fatigue represents fatigue “caused by mechanisms distal to the neuromuscular junction” (Staud, 2012). Therefore, central fatigue is related to the failure of motivational and affective input, leading to a higher perception of effort and resulting in the sense of fatigue (Staud, 2012). Peripheral fatigue includes multiple neuromuscular changes affecting the contractility of muscles (Staud, 2012). The major mechanisms involved in peripheral fatigue include changes in activation of the muscle such as: impaired action potential propagation along the sarcolemma, reduced SR Ca^2+^ release and reuptake, decreased Ca^2+^ sensitivity of the myofibrils, and decreased number of force-generating crossbridges or force produced per crossbridge (Allen et al., 2008; Enoka and Duchateau, 2008). Peripheral fatigue arises from impaired EC coupling that leads to decreased force produced by the contracting muscle. In order to increase force production, there is a need for increased motor neuron pool activation via an increased cortical drive. Increased cortical drive can lead to a higher perception of effort, which in turn decreases exercise tolerance and contributes to fatigability. Therefore, both central and peripheral mechanisms of fatigue are not necessarily mutually exclusive (Gandevia, 2001; Pageaux, 2016).

There are key limitations to the central-peripheral fatigue continuum: (1) the belief that the mechanisms needed to maintain task performance while counteracting declining muscle force capacity are independent from those generating sensations of fatigue, and (2) during experimentation, almost all of the physiological processes in a task-dependent action, from the CNS to the muscle involved, can contribute to fatigue, implying that it would be very difficult to distinguish all the potential contributors to fatigue without a holistic perspective (Enoka and Duchateau, 2016). In order to move away from the central-peripheral dichotomy, a new taxonomy involving the term “fatigability” has been suggested (Kluger et al., 2013; Enoka and Duchateau, 2016). While the term fatigue describes a “state of exhaustion and decreased strength”, the expression fatigability denotes “susceptibility to fatigue” (Nikolaus et al., 2013; Enoka and Duchateau, 2016). In other words, individuals who are less fatigable reach the same level of fatigue as others at a much greater demand (Enoka and Duchateau, 2016). Perceptions of fatigue, also referred to as the subjective sensations of fatigue, and fatigability are distinct and possibly independent, but establishing an association between the two is very important. “Perceptions of fatigue” and “performance fatigability” are subsections of fatigue that each contain further categories of factors related to fatigue. “Perceptions of fatigue” contain homeostatic and psychological factors, such as central regulation based on feedback or depression, whereas “performance fatigability” includes peripheral factors and central factors, similar to the mechanisms involved in peripheral, and central fatigue (Kluger et al., 2013; Enoka and Duchateau, 2016).

Further revisions to the taxonomy now include the current extent of conditions associated with fatigue. The concept of fatigue in this new taxonomy includes two attributes: performance fatigability and perceived fatigability. Performance fatigability regards “the decline in an objective measure of performance over a discrete period” where perceived fatigability includes “changes in sensations that regulate the integrity of the performer” (Enoka and Duchateau, 2016). A key feature of the taxonomy is that the degree of fatigue that limits both physical and cognitive function in individuals varies due to the many factors of performance and perceived fatigability within and between each category. Perceived fatigability still refers to homeostatic processes and psychological state, where performance fatigability refers to contractile function and muscle activation (Enoka and Duchateau, 2016). Perceived fatigability in an individual can be evaluated at both rest or during physical activity, where performance is evaluated through strenuous activity (Staud, 2012; Enoka and Duchateau, 2016).

Reporting fatigue should be focused on stating the primary outcome variable. Perceived fatigability can be measured through the use of subjective surveys to individually rate various dimensions of fatigue (Kluger et al., 2013; Enoka and Duchateau, 2016). Where performance fatigability can be measured through fatigue-inducing tasks, perceived fatigability should be measured though the individual’s interpretation of their fatigue levels (Enoka and Duchateau, 2016). Assessments of performance fatigability focus on an outcome variable that can be evaluated throughout the duration of a given task (Enoka and Duchateau, 2016). The ability to complete physical tasks is quantified with clinically and laboratory-based methods testing endurance, strength, manual dexterity and locomotion. Such measures of fatigue include the completion time of specific tasks, the duration a fatigue-inducing task can be sustained, and the rate of change in variables associated with human performance (Enoka and Duchateau, 2016). Maximum voluntary isometric contraction (MVC) force, which indicates the greatest amount of tension a muscle can generate or hold, can also be measured objectively through various laboratory techniques such as through time to task failure (Enoka and Duchateau, 2016). Walking endurance or time trials can be used when measuring the completion time of specific tasks. Results from laboratory studies testing human performance demonstrate the severity of fatigue an individual endures after or during completion; for instance, a shorter time to task failure would indicate increased fatigability in a patient compared to healthy controls who are less susceptible to fatigue and are able to sustain the activity for a longer period of time.

Throughout the literature, it is apparent that the use of subjective measures of fatigue (surveys and self-reports) are more common than objective measures in RA populations. There are twelve patient-reported outcome measures (PROMs) that have been most recently utilized to measure fatigue in RA (Hewlett et al., 2011). Of twelve fatigue scales reviewed within the literature, five were specific to RA. The five scales include Bristol Rheumatoid Arthritis Fatigue Multi-Dimensional Questionnaire, Bristol Rheumatoid Arthritis Fatigue Numerical Rating Scales (severity, effect and coping), Functional Assessment Chronic Illness Therapy (Fatigue), Multi-Dimensional Assessment of Fatigue, and Multi-Dimensional Fatigue Inventory (Hewlett et al., 2011). Each PROM involves questions specific to fatigue and requires individuals to rank their level of fatigue on a scale – it could be their current fatigue levels, or fatigue levels over a certain amount of time (Hewlett et al., 2011; Nikolaus et al., 2013). Validity evidence for each PROM differs, as demonstrated in **Table 2**. Content validity refers to how well a test measures the symptom for which it is intended, construct validity refers to the degree to which a test measures what it claims to be measuring and criterion validity refers to the extent to which a measure is related to an outcome (Hewlett et al., 2011). Hewlett et al. noted that because fatigue is experienced as a multi-dimensional symptom with short-term or long-term duration, relying on PROMs alone can be difficult (Hewlett et al., 2011; Nikolaus et al., 2013). Specifically, it is the unpredictable multifactorial nature of fatigue in chronic inflammation that makes it difficult to evaluate fatigue using PROMs (Hewlett et al., 2011; **Table 2**).

**Table 2:** Patient-reported outcome measure scales developed with, evaluated in or specific to Rheumatoid Arthritis (Hewlett et al., 2011).

Scale	Content	Validity
Bristol Rheumatoid Arthritis Fatigue Multi-Dimensional Questionnaire	Measures impact and severity of fatigue over the previous 7 days in rheumatoid arthritis	Content validity: strong
		Construct validity: strong
		Criterion validity: strong
Bristol Rheumatoid Arthritis Fatigue Numerical Rating Scales (severity, effect and coping)	Measures impact, coping and level of fatigue over the previous 7 days in rheumatoid arthritis patients	Content validity: strong
		Construct validity: strong
		Criterion validity: strong,
		moderate for coping
Functional Assessment Chronic Illness Therapy (Fatigue)	Measures fatigue levels in chronic illness over the previous 7 days	Content validity: moderate
		Construct validity: strong
		Criterion validity: strong
Multi-Dimensional Assessment of Fatigue	Measures severity, distress, timing and impact of fatigue over the previous 7 days	Content validity: moderate
		Construct validity: strong
		Criterion validity: strong
Multi-Dimensional Fatigue Inventory	Measures general, physical and mental fatigue as well as reduced motivation and activity	Content validity: moderate
		Construct validity: strong
		Criterion validity: moderate

Throughout this review, we advocate that fatigue should be assessed as a symptom, highlighting the need for subjective measures (Enoka and Duchateau, 2016). The more fatigue an individual experiences, the more difficult the task is perceived by the patient, which decreases motivation to continue or complete the task (Staud, 2012). In order to grasp an appreciation for self-reported fatigue, it may be necessary to complete both objective and subjective measures in clinical research in order to truly compare how both measures correspond to one another. This can determine if subjective measures used to document perceived fatigability truly correlate with objective measures of performance fatigability. Results from a recent study, indicated that neuromuscular fatigue during a 60 s isometric contraction, was weakly associated with RA patients’ perceptions of fatigue (do Espírito Santo et al., 2017). It is important to note that performance fatigability is highly task-dependent (Enoka and Duchateau, 2016), thus it is not known whether perceptions of fatigue in this RA population better relate to dynamic fatiguing contractions such as those experienced during everyday life. This finding provides a proof of concept in linking perceived and performance fatigability in future clinical studies with the aim to fully capture the extent to which fatigue cognitively and physically impairs an RA patient’s ability to function in everyday life (do Espírito Santo et al., 2017).

## Training Studies: Exercise and Perceived Barriers

There are various symptoms associated with RA that patients regard as their top priorities for treatment: less fatigue, less pain, hindering joint damage, ability to carry out activities of daily living, and improved mobility (Chauffier et al., 2011). Recent research indicates that exercise and physical activity can improve these disease-related symptoms as well as enhance mental health in RA patients (Veldhuijzen Van Zanten et al., 2015). The completion of both resistance training and endurance training in RA patients offers many benefits in skeletal muscle, bone, and joint health (Cooney et al., 2011). Patients with RA can benefit from the anabolic effects of exercise similarly to individuals without RA (Cooney et al., 2011). Progressive resistance training (PRT) has been demonstrated to increase muscle mass, strength, and function in RA patients, reducing and managing the risk of cachexia and sarcopenia (Masuko, 2014). PRT also improves bone mineral density, as well as benefits tendons and connective tissue (Cooney et al., 2011). Endurance training (ET) is also very important in increasing insulin sensitivity, improving body composition, and decreasing circulating CRP and IL-6 levels (Powers et al., 1999). Muscle antioxidant capacity is also increased following ET (Powers et al., 1999; Reeves et al., 2003) which may help counterbalance the chronically elevated redox stress in skeletal muscle of RA patients. The combination of both PRT and ET therefore helps to increase lean body mass, decrease body fat and improve disease activity in RA patients (Masuko, 2014). Importantly, both aerobic and resistance training also reduce the self-reported levels of fatigue in individuals with RA (Cooney et al., 2011; Cramp et al., 2013). Based on these studies and additional evidence brought forward by a systematic review regarding the effect of dynamic exercise programs on RA, it is evident that ET and PRT have positive effects on aerobic capacity and muscle strength, and should thus be recommended as a routine intervention for patients with RA (Hurkmans et al., 2009).

Even though exercise has been recommended as part of the management and treatment of RA and its co-morbidities, 71% of RA patients do not participate in regular physical activity (Veldhuijzen Van Zanten et al., 2015). Low physical activity levels indicate that many individuals with RA perceive various barriers when attempting exercise even if they are aware of the benefits. The primary barriers identified are disease-related, self-reported pain, and fatigue (Veldhuijzen Van Zanten et al., 2015). By overcoming perceived fatigability and motivating oneself to participate in physical activity, it is possible that performance fatigability can be reduced as well. Furthermore, by reducing performance fatigability, perceived fatigability is also potentially decreased, thus improving an individuals’ perception that they are able to overcome various barriers (Veldhuijzen Van Zanten et al., 2015). In numerous training studies it has been documented that exercise can significantly reduce the sensation of fatigue (Cramp et al., 2013) as well as the pathogenesis of RA (Matschke et al., 2010b), which highlights the need for physical activity to be part of the treatment for managing symptoms in RA. A common misconception in individuals with RA is that intense exercise will further damage their joints (Cooney et al., 2011), yet, a recent meta-analysis reported that two of four studies investigating physical activity as an intervention for RA determined a statistically significant reduction in joint tenderness and swelling (Cramp et al., 2013). There is a clear need for clinicians and rehabilitation professionals to educate RA patients on the potential benefits of physical activity in alleviating and managing disease symptoms Halls et al. (2017). Although current literature suggests that among patients with RA, ET should be conducted at 60–85% of maximum heart rate (HR) for 30–60 min per week, and PRT should be conducted at 50–80% of the maximal voluntary contraction 2–3 times per week (Stenstrom and Minor, 2003), further research is still required to determine the optimal dose and types of exercise that can provide the best clinical outcomes for RA patients (Cooney et al., 2011; Veldhuijzen Van Zanten et al., 2015). Furthermore, future studies are needed to determine valid measurement methods of true baseline strength and fatigue as these factors will vary greatly between individuals due to perceived fatigability, pain, and disease status (Stenstrom and Minor, 2003).

## Future Directions

There is still conflicting data within the literature regarding fatigue and the ways in which to evaluate fatigue within various populations. In order to make the literature on fatigue more coherent, we should move away from terms describing the origin of fatigue and instead focus on the terms “perceived fatigability” and “performance fatigability” as subsets of fatigue that are involved with both central and peripheral factors related to the symptom. Approaching the term fatigue as a symptom is necessary for the future foundation of measuring fatigue (Enoka and Duchateau, 2016). Self-reports are insufficient to measure performance fatigability, so instead we should focus on validated outcome measures of human performance in order to quantify performance fatigue levels (Enoka and Duchateau, 2016). The multifactorial and subjective nature of fatigue demonstrates the need for determining a multi-module approach to the evaluation of fatigue in healthy and rheumatoid populations (Hewlett et al., 2011).

## Summary

Overall, there is still confusion in the literature regarding fatigue, fatigability, and the best tools to measure fatigue, especially in RA patients. Fatigue is influenced by two domains: performance fatigability and perceived fatigability (Kluger et al., 2013; Enoka and Duchateau, 2016). Performance fatigability indicates the factors that lead to a measurable decline in performance over a specific period of time, such as contractile function and muscle activation, where perceived fatigability indicates the subjective state of the individual relating to psychological factors and deviation from homeostasis within the body (Enoka and Duchateau, 2016). Future studies should attempt to include both measures in order to correlate objective and subjective fatigue in their subjects/patients, as a limited number of clinical studies have included both in the past. Based on the latest evidence, it is apparent that evaluation of perceived fatigability would include the use of self-reports, PROMs, while evaluating performance fatigue would include clinical/laboratory assessments of outcome measures specific to human performance in order to gain accurate representation (Hewlett et al., 2011; Enoka and Duchateau, 2016; do Espírito Santo et al., 2017). There is a need for the development of fatigue measures specific to inflammatory diseases. However, the fluctuating nature of perceived fatigue in these populations, from persistent fatigue to overwhelming events of fatigue, makes it difficult to gain accurate measures of the symptom (Hewlett et al., 2011). Further research is needed to address a multi-module approach to measuring fatigue in these populations in order to differentiate fatigue developed from the disease itself compared to normal physiological and aging processes, understand the concomitant nature of performance fatigability and perceived effort, as well, identify appropriate therapeutic interventions to minimize fatigue in patients with RA.

## Search Methods

The following electronic databases were searched: PubMed database (1985 to present) and Cochrane Database of Systematic Reviews (2000 to present). In addition, we searched the reference lists of key articles, both human and animal studies, and review articles. We combined key words associated with the term ‘fatigue’ that were of relevance to our review (fatigability, rheumatoid arthritis, performance fatigability, perceived fatigability, and neuromuscular).

## Author Contributions

All authors contributed to the conception and design of the review and drafting the manuscript.

## Conflict of Interest Statement

The authors declare that the research was conducted in the absence of any commercial or financial relationships that could be construed as a potential conflict of interest.
